# The conserved wobble uridine tRNA thiolase Ctu1 is required for angiogenesis and embryonic development

**DOI:** 10.1371/journal.pone.0315854

**Published:** 2024-12-20

**Authors:** Yangziwei Yu, Chuqiao Wang, Yan Wang, Heng Shi, Huiyuan Hu, Yibin Du, Zhaoli Zhou

**Affiliations:** 1 School of Medical Instrument and Food Engineering, University of Shanghai for Science and Technology, Shanghai, China; 2 Collaborative innovation Center for Biomedicine, Shanghai University of Medicine and Health Sciences, Shanghai, China; 3 Department of Pharmaceutical Toxicology, School of Pharmacy, China Medical University, Shenyang, China; 4 Shanghai University of Traditional Chinese Medicine, Shanghai, China; 5 Shanghai World Foreign Language Academy, Shanghai, China; 6 Jiading District Central Hospital Affiliated Shanghai University of Medicine and Health Sciences, Shanghai University of Medicine and Health Sciences, Shanghai, China; University of Colorado Boulder, UNITED STATES OF AMERICA

## Abstract

Cytosolic thiouridylase is a conserved cytoplasmic tRNA thiolase composed of two different subunits, CTU1 and CTU2. CTU2 serves as a scaffold protein, while CTU1 catalyzes the 2-thiolation at the 34^th^ wobble uridine of the anticodon loop. tRNAGln^UUG^, tRNAGlu^UUC^, and tRNALys^UUU^ are the tRNA substrates that are modified with a thiol group at the C2 positions (s^2^) by CTU1, and also with a methoxycarbonylmethyl group at the C5 positions (mcm^5^) by Elongator and ALKBH8. mcm^5^s^2^U_34_ modification of the three tRNAs, and their modifying enzymes are involved in human disease and development. Elongator mutant animals exhibit severe phenotypes, while the biological function of Ctu1 in vertebrate animal models remains poorly characterized. Here, we applied antisense morpholino oligonucleotides targeting *cytosolic thiouridylase subunit1* (*ctu1)* transcripts in a zebrafish model and small interfereing RNA against CTU1 transcript in human endothelial cells to define the phenotypes. We found that deficiency of *ctu1* causes impaired angiogenesis and development in zebrafish embryos, and CTU1 is involved in proliferation, migration, and tube formation of human endothelial cells. We employed single-cell RNA sequencing to acquire the transcriptomic atlas from *ctu1* and control morphant zebrafish. Comprehensive bioinformatics analysis, including pseudo-time, RNA velocity, cell-cell communication, and gene regulatory network inference revealed that *ctu1* deficiency leads to the arrest of cell cycle, and the defects of nerve development and erythrocyte differentiation and the attenuation of several pro-angiogenic signaling pathways, e.g., *angpt-tek* and *dll4-notch*. Our findings show for the first time that CTU1 is essential for angiogenesis and embryonic development in vertebrates.

## Introduction

tRNA undergoes extensive post-transcriptional chemical modifications in the nucleotides of the anticodon loop [1]. In eukaryotes, the cytosolic tRNAGln^UUG^, tRNAGlu^UUC^, and tRNALys^UUU^ are the three types of tRNA that have uridine at the wobble position 34 (U_34_). The U_34_ of these tRNAs is chemically modified with a thiol group at the C2 positions (s^2^), and (commonly) a methoxycarbonylmethyl group at the C5 positions (mcm^5^), which ultimately results in a mcm^5^s^2^U_34_ [2, 3]. The mcm^5^s^2^U_34_ modification occurs through two pathways. In one pathway, *urm1* acts as a sulfur donor for cytosolic tRNA thiouridylase subunit 1 (CTU1), which catalyzes the thiolation of cytosolic tRNAGln^UUG^, tRNAGlu^UUC^, and tRNALys^UUU^ [4]; while the other one consists of ALKBH8 (alkB homolog 8, tRNA methyltransferase) and the Elongator complex (ELP1-ELP6), which participate in the formation of the mcm^5^ side chain [5].

mcm^5^s^2^U_34_ modification and its modifying enzymes have been regarded as critical regulators of gene expression and protein homeostasis [6]. mcm^5^s^2^U_34_ modifying enzymes play crucial roles in many important physiological and pathological processes [7, 8]. Several clinical cases indicate that mutations in the mcm^5^s^2^U_34_ modifying enzymes, like Elongator subunits (ELP2, ELP4, and ELP6), ALKBH8 and CTU2, are associated with human neurological diseases [9–11]. Elp2 mutant mice exhibit a severe neuro developmental phenotype [12], and Elp3 knock-out mice show embryonic lethality [13]. Double deletion of the partner enzymes (CTU1 and ELP3) for mcm^5^s^2^U_34_ modification is lethal to the cell in yeast [14]. Single deletion of the s^2^U_34_ modification enzyme (Ctu1 or Ctu2) in the nematode and yeast causes development abnormalities, thermosensitivity [15], and sensitivity to various exogenous stresses [16, 17]; however, the *in vivo* role of Ctu1 in vertebrates, and the underlying mechanisms remain to be clarified.

Single-cell RNA sequencing (scRNA-seq) technology enables the unraveling of genetic and transcriptional heterogeneity among cells and provides novel insights into gene function [18]. In this study, we employed morpholino (MO) antisense oligonucleotides targeting *ctu1*, and scRNA-seq in zebrafish embryos to define the phenotypes and the single-cell transcriptomic atlas in response to *ctu1* deficiency. We also applied lentivirus particles in human endothelial cells to establish CTU1 overexpression (CTU1-OE) and CTU1 knockdown (CTU1-KD) endothelial cells, and examined their angiogenesis-related behaviors. Our data demonstrated that CTU1 plays a vital role in proliferation and development, and also in nerve and erythrocyte differentiation. Several pro-angiogenic signaling cascades, e.g., *angpt-tek* and *dll4-notch*, are the potential molecular mechanisms underlying the *ctu1*-related angiogenesis defects.

## Materials and methods

### Maintenance and genetic manipulation of zebrafish *(Danio rerio)*

All zebrafish experiments were carried out with the approval of the Animal Care and Use Committee of Shanghai University of Medicine and Health Sciences, and followed the regulations specified in EU Directive 2010/63/EU. Tg*(fli1a-EGFP;casper)* lines of zebrafish were maintained in the facility of the Shanghai Research Center for Model Organisms. Antisense MO was designed by Gene Tools (http://www.gene-tools.com/), and microinjected into fertilized one-cell stage embryos at 4 ng using MN-151 micromanipulator (Narishige, Japan) according to standard protocols [19]. Antisense MO targeting the intron 2-exon 3 splice of *ctu1* was 5’-GCATTGTGACCTGCTCATCAAACAA-3’. Antisense MO targeting the exon 2-intron 2 of *urm1* was 5’-GAATACTTTTCCATACTCACAGGGA-3’. The standard control MO sequence was 5’-CCTCTTACCTCAGTTACAATTTATA-3’. All zebrafish embryos employed in this study were without sexual differentiation.

### Zebrafish embryonic angiogenesis

After MO microinjection, embryos were dechorionated at 2 days post fertilization (2-dpf), and anesthetized with 0.016% MS-222 (tricaine methanesulfonate, Sigma-Aldrich, St. Louis, MO). Zebrafish larvae were then oriented on lateral side (anterior, left; posterior, right; dorsal, top), and mounted with 3% methylcellulose in a depression slide for observation and photography by fluorescence microscopy with digital cameras (Nikon SMZ 1500). A subset of images was adjusted for levels, brightness, contrast, hue and saturation with Adobe Photoshop 7.0 software (Adobe, San Jose, California) to optimally visualize the expression patterns. Quantitative image analyses processed using image based morphometric analysis (NIS-Elements D3.1, Japan) and ImageJ software (U.S. National Institutes of Health, Bethesda, MD, USA; http://rsbweb.nih.gov/ij/).

### Cell culture and lentivirus infection

Human microvascular endothelial cells (HMEC-1) (SUNNCELL, Shanghai, China) were cultured in MCDB131 medium supplemented with 10 ng/mL epidermal growth factor (Thermo, USA), 1 μg/mL hydrocortisone (Sigma, Germany), 10 mM L-Glutamine (Gibco, New York, USA), 10% FBS (Gibco, USA), and 1% penicillin-streptomycin solution (Gibco, USA), and kept in an incubator (Thermo, USA) at 37°C, with 5% CO_2_. The lentiviral vectors pSLenti-U6-sh(CTU1)-CMV-EGFP-F2A-Puro-WPRE, pSLenti-U6-CMV-EGFP-F2A-Puro-WPRE, and pSLenti-CMV-Ctu1-3xELAG-PGK-Puro-WPRE were constructed, and packaged into lentivirus particles by OBiO Technology (OBiO, Shanghai, China). Cells were infected with the lentivirus and 10 μg/ml Polybrene at 37°C. Then the cells were seeded into a 96-well plate (7000–8000 cells per well) and the cell viability was determined by Cell Counting Kit-8 (CCK-8, Beyotime, China) after 24, 48, and 72 hours’ culture.

### RNA isolation and PCR analysis

Total RNA was extracted using the RNA-easy Isolation Kit (Vazyme Biotech, China) following the manufacturer’s guidelines. Subsequently, 1 μg of the isolated RNA was reverse transcribed using the HiScript^®^ III All-in-one RT SuperMix Perfect for qPCR kit (Vazyme Biotech, China). Using the SYBR green reaction mix (Vazyme Biotech, China, #Q711-02), a QuantStudio^™^ 5 Real-Time PCR System (Cell Signaling Technology, MA) was utilized to conduct a quantitative PCR analysis. For the RT-PCR analysis of the gene expression after MO injection, primers spanning *ctu1* exon 2 and exon 3 were used as 5’-GCAGGTGGGCTTGAAGAATAACT-3’ (forward), and 5’-GTGCAGCGGCGGAGACGAG-3’ (reverse) for the *ctu1* I2E3-MO; primers spanning *urm1* exon 1 and exon 3 were used as 5’-TGAGATTACTTCCGGGTTTTACAA-3’ (forward) and 5’-GTGCAGCGGCGGAGACGAG-3’ (reverse) for *urm1* e2i2-MO. The primer *ef1α* sequences used as the internal control were 5’-GCTCTGGGCGCTCCTTTAG-3’ (forward) and 5’-GATACCAGCCTCAAACTCACC-3’ (reverse). The primer sequences for human CTU1 were 5’-GGTCGTGGCCTACGAAGA-3’ (forward) and 5’-AGTTCATGAGCACGGTCTCC-3’ (reverse). The primer sequences for the internal control human gene gene ACTB were 5’-TGGCACCACACCTTCTACAA-3’ (forward) and 5’-CCAGAGGCGTACAGGGATAG-3’ (reverse).

### Transwell assays

Transwell assays were conducted with the 24-well plate (Corning, USA) according to manufacturer’s instructions. In brief, after 12 hours of starvation, 1.5 × 10^4^ HMEC-1 cells were seeded in the upper chambers of the inserts, with the culture medium supplemented with 20% FBS used in the lower chambers. After 24 h of incubation at 37°C and removal of those non-invasive cells, the remaining cells were then fixed and stained with 1% crystal violet for 10 minutes at room temperature. Finally, those migrated cells were visualized and quantified through microscopic examination at a magnification of ×200 (Leica, Germany).

### Tube formation assays

The matrigel-based tube formation assay were conducted following a previously established protocol [20]. In brief, a 50 μL layer of Matrigel (Corning, New York, USA) was evenly coated in a 96-well plate, then 1.5 × 10^4^ HMEC-1 cells were seeded and incubated at 37°C with 5% CO_2_ for 16 h. Then, the formed tubes were observed and photographed under a microscope (Eclipse Ts2R; Nikon, Japan). ImageJ software (National Institutes of Health, USA) was used for the quantification of the branch points, tube branch length, and total covered area.

### Single-cell library preparation

Single cell preparation, library construction, and sequencing were performed by Sinotech Genomics Co., Ltd. Shanghai, China. In brief, about 20 zebrafish fertilized eggs at 2-dpf were collected from control and *ctu1* morphant. Following the digestion process as before [21], cell pellets were collected, and finally loaded as a single-cell suspension into the BD Rhapsody system (BD Biosciences, San Jose, CA). The cDNA library was generated from double strand full length cDNA by random priming amplification with the BD Rhapsody cDNA Kit (#633773, BD Biosciences) and the BD Rhapsody Targeted mRNA & AbSeq Amplification Kit (#633774, BD Biosciences). All the libraries were sequenced in a PE150 mode (Pair-End for 150bp read) in the X Ten instrument (Illumina, San Diego, CA).

### scRNA-seq data processing and quality control

The BD Rhapsody Whole Transcriptome Assay Analysis Pipeline was used. The FASTQ documents were filtered to generate a single cell expression profile matrix. The R software (version 4.2.2) and Seurat (version 4.3.0) were utilized for downstream clustering and visualization. After filtering cells with unique features over 2,000 or less than 200 and mitochondrial counts exceeding 20%, we normalized the data with the “LogNormalize” method. Subsequently, the “RunPCA” function was used for Principal Component Analysis (PCA). Clustering analysis was then conducted utilizing the “FindClusters” function. For visualization, Uniform Manifold Approximation and Projection (UMAP) coordinates were determined through the “RunUMAP” function. The marker genes of each cluster were calculated by the “FindAllMarkers” function with log2FC.threshold = 0.25; min.pct = 0.25.

### Cell cycle, differential expression, and gene set enrichment analysis (GSEA)

Initially, zebrafish genes were transformed into human genes using the “homologene” function. Subsequently, the cell cycle state was characterized using the cell cycle-related signature sets incorporated in the Seurat package and the “CellCycleScoring” function. Ultimately, the cell cycle phase was labeled. The differentially expressed genes (DEGs) were identified by calculating the “FindMarkers” function with min.pct = 0.25, log2FC.pct = 0.25. Genes with an absolute log_2_FC greater than 0.5 and an adjusted p-value less than 0.05 were considered as DEGs with significant difference between the two groups. GSEA of the DEGs were also performed by R package clusterProfiler (version 4.2.2). The “gseKEGG” and “gseGO” functions for analysis were used with OrgDb = "org.Dr.eg.db".

### Pseudo-time analysis

Monocle 2, version 2.22.0 was used to determine the pseudotemporal ordering of erythroid cells. Briefly, erythroid cells of wild type (WT) and Ctu1 morphant were subclustered and a cellDataSet object was created in Monocle2 with the function “newCellDataSet” with standard arguments enabled. Genes with an average expression value greater than 0.1 were used to sort the cells in the quasi-time trajectory. Dimensionality reduction was conducted using discriminative dimensionality reduction with trees (DDRTree). Erythroid cells were ordered in pseudotime using the “orderCells” function. The dynamic expression of genes was visualized using the “plot_genes_in_pseudotime” function.

### RNA velocity analysis

The ratio between unspliced and spliced mRNA levels can predict the subsequent concentration of mature mRNA, which subsequently forecasts the future cellular state [22]. The spliced and unspliced reads of scRNA-seq data were calculated based on the bam file and saved the results as loom files. Loom files for individual libraries from control and Ctu1 morphant, were combined using loompy, version 2.0.16. The scVelo, version 0.2.2 was employed to estimate the velocity vector based on the RNA velocity dynamic model with default parameters, assess the transition probability matrix in a dynamic system, infer alterations in cell state over time, and map the RNA velocity results onto UMAP images.

### Cell-cell communication analysis

CellChat version 1.6.1 was used to analyze intercellular communication networks [23]. We performed sub-clustering and normalization of mesoderm cells as input data for CellChat to infer the cell-cell communication networks. The “homologene” function was used for cross-species homologous gene conversion. The significant ligand-receptor interactions were identified using the “identifyOverExpressedInteractions” (*p* = 0.05) function. The function “computeCommunProb” was utilized to ascertain the probability of intercellular communication, with the parameters set to raw.use = FALSE, nboot = 100, Hill function parameter kn = 0.5. The function“computeCommunProbPathway” was employed to infer potential communication pathways between cells, using a threshold of 0.05. The cell-cell communication networks were calculated via the aggregateNet function, also with a threshold of 0.05.

### Gene regulatory network (GRN) inference

To infer the GRN between control and Ctu1 morphant, the SCENIC analysis was conducted utilizing the pySCENIC software [24]. After quality control and homologous gene conversion in Seurat, the normalized data was exported to a matrix and then converted into a loom file. A list of human transcription factors (TFs) (https://github.com/aertslab/pySCENIC/blob/master/resources/hs_hgnc_tfs.txt) was utilized and regulatory interactions between them and their potential target genes were inferred using GRNBoost2. CisTarget motif enrichment was conducted using SCENIC’s RcisTarget and ranking databases (hg19-tss-centered-10kb-7species.mc9nr.genes_vs_motifs.rankings.feather). The activity of the regulons was computed using SCENIC’s “AUCell” function. The activity of regulons in cells under different conditions is determined by Regulon Specificity Scores (RSS) and Z-scores [25].

### Statistical analysis

Statistical analysis were performed using R software and GraphPad Prism software. The difference between the two groups was analyzed using the Wilcoxon signed-rank test or t-test. For comparisons involving three or more groups, the Kruskal-Wallis test was employed. Unless otherwise specified, statistical significance was defined as *p* < 0.05.

## Results

### Phenotype resulting from *ctu1* deficiency in zebrafish embryo

**Fig 1:**
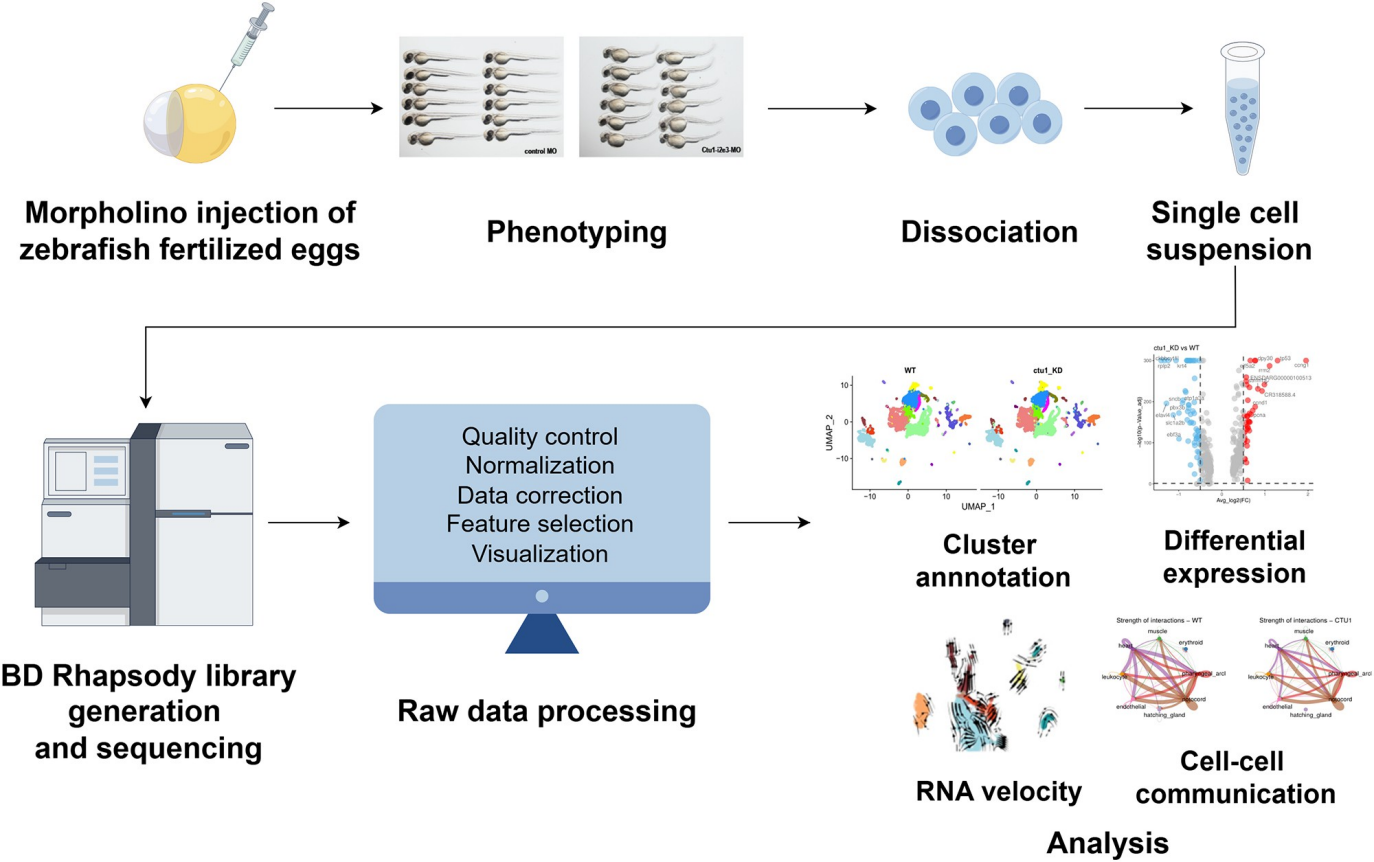
Schematic representation of workflow, figured (by Figdraw https://www.figdraw.com/). Antisense MO oligonucleotides were microinjected into fertilized one-cell stage embryos. At 2-dpf, zebrafish larvae were either photographed for phentying, or collected for single-cell preparation and sequencing using BD Rhapsody system. After filtering the raw data, further single-cell data analysis was performed such as cluster annotation, DEGs analysis, RNA velocity, cell-cell communication.

**Fig 2:**
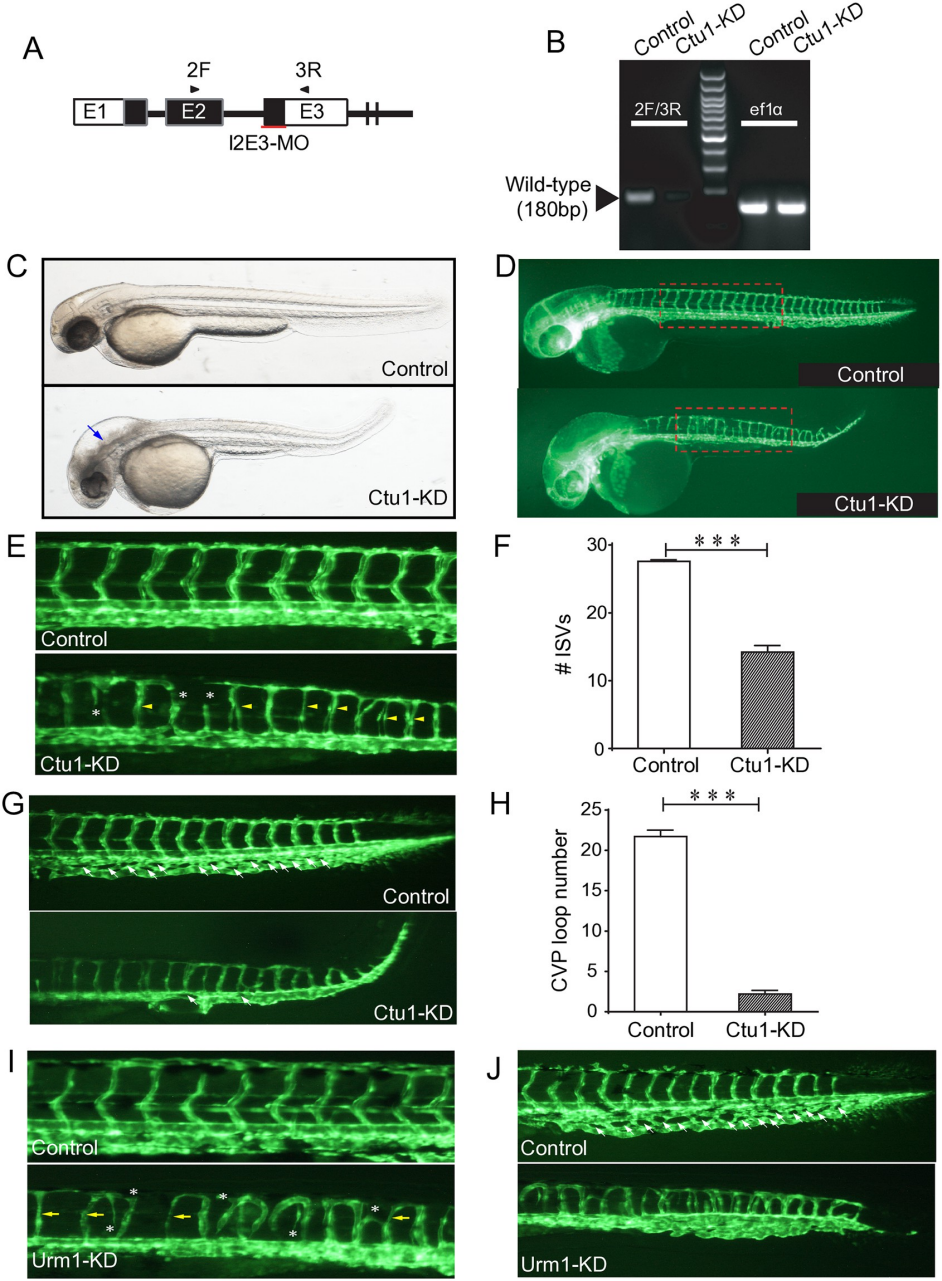
*ctu1* morphant zebrafish larvae exhibits developmental defects. (A) Ctu1-targeted MO design strategy. (B) PCR analysis of control and Ctu1 morphant. (C and D) Bright-fieldand EGFP fluorescentimages depict the overall morphology of control and Ctu1 morphant at 2-dpf. Blue arrows indicate expanded brain ventricle and hindbrain edema in *ctu1* morphant compared with control. The dotted square regions are shown at higher magnification in E. (E and G) Image of trunk regions. Compared with control MO, embryos injected with *ctu1*-i2e3-MO present a lower number of incomplete and thinner intersegmental vessels (ISVs, yellow arrows), and ectopic sprouts (asterisk) of dorsal aorta (E, lower panel). In control embryos, caudal vein plexus (CVP, white arrows) were formed honeycomb-like structures at the tail around 2-dpf (G, upper panel, arrowheads). In contrast, *ctu1* deficency resulted in specific defects in CVP formation (G, lower panel, arrowheads). Quantification of the number of complete ISVs (F) and CVP (H). Columns, mean; bars, SEM (n = 10; unpaired student’s t-test; ***, *p <* 0.001). (I and J) Image of trunk regions. Compared with control MO, embryos injected with *urm1*-i2e2-MO present a lower number of incomplete and thinner ISVs (yellow arrows), and ectopic sprouts (asterisk) of dorsal aorta (I, lower panel). In control embryos, CVP (white arrows) were formed honeycomb-like structures at the tail around 2-dpf (J, upper panel, arrowheads). In contrast, *urm1* deficency resulted in specific defects in CVP formation (J, lower panel, arrowheads).

The workflow of our study was illustrated as Fig 1. Tg*(fli1a*:*EGFP)*y1 zebrafish line is an ideal model for visualizing the embryonic vasculature formation [26]. Its embryo is transparent, and its endothelial cells were green fluorescent labeled. We applied Tg*(fli1a*:*EGFP)*y1 zebrafish line, and targeted the expression of *ctu1* by specific MO antisense strategies (Fig 2A). Two days after the MO injection, nucleic acids were extracted from the embryos for RT-PCR analysis. The PCR data revealed that the antisense Ctu1-i2e3-MO effectively knocked down *ctu1* expression (Fig 2B). Representative bright field and fluorescence image analysis revealed that *ctu1* morphant embryo showed a developmental abnormalities and defective angiogenesis. The *ctu1* morphant embryos display significant morphological abnormalities including enlarged brain ventricles, pronounced hindbrain edema, pericardial edema, a misshapen spine, and a curled-up tail (Fig 2C and 2D, S1A and S1B Fig). Meanwhile, the *ctu1* morphant exhibit a reduced number of incomplete and thinner intersegmental vessels (ISVs), dorsal longitudinal anastomotic vessels (DLAV), caudal vein plexus (CVPs), ectopic sprouts of dorsal aorta (DA) and posterior cardinal vein (Fig 2E–2H). Meanwhile, as Urm1 acts as a sulfur donor for Ctu1 in the thiolation of cytosolic tRNAGln^UUG^, tRNAGlu^UUC^, and tRNALys^UUU^, we also examined the effects of MO-mediated down-regulation of *urm1* (S1C and S1D Fig). The phenotypes in *urm1* morphant are quite similar as the ones in *ctu1* morphant (Fig 2I and 2J, S1E–S1H Fig). The result further strengthens the evidence supporting that the Ctu1 is required for vascular networks and embryonic development.

### Single-cell transcriptome atlas of the ctu1 morphant and control zebrafish embryos

**Fig 3:**
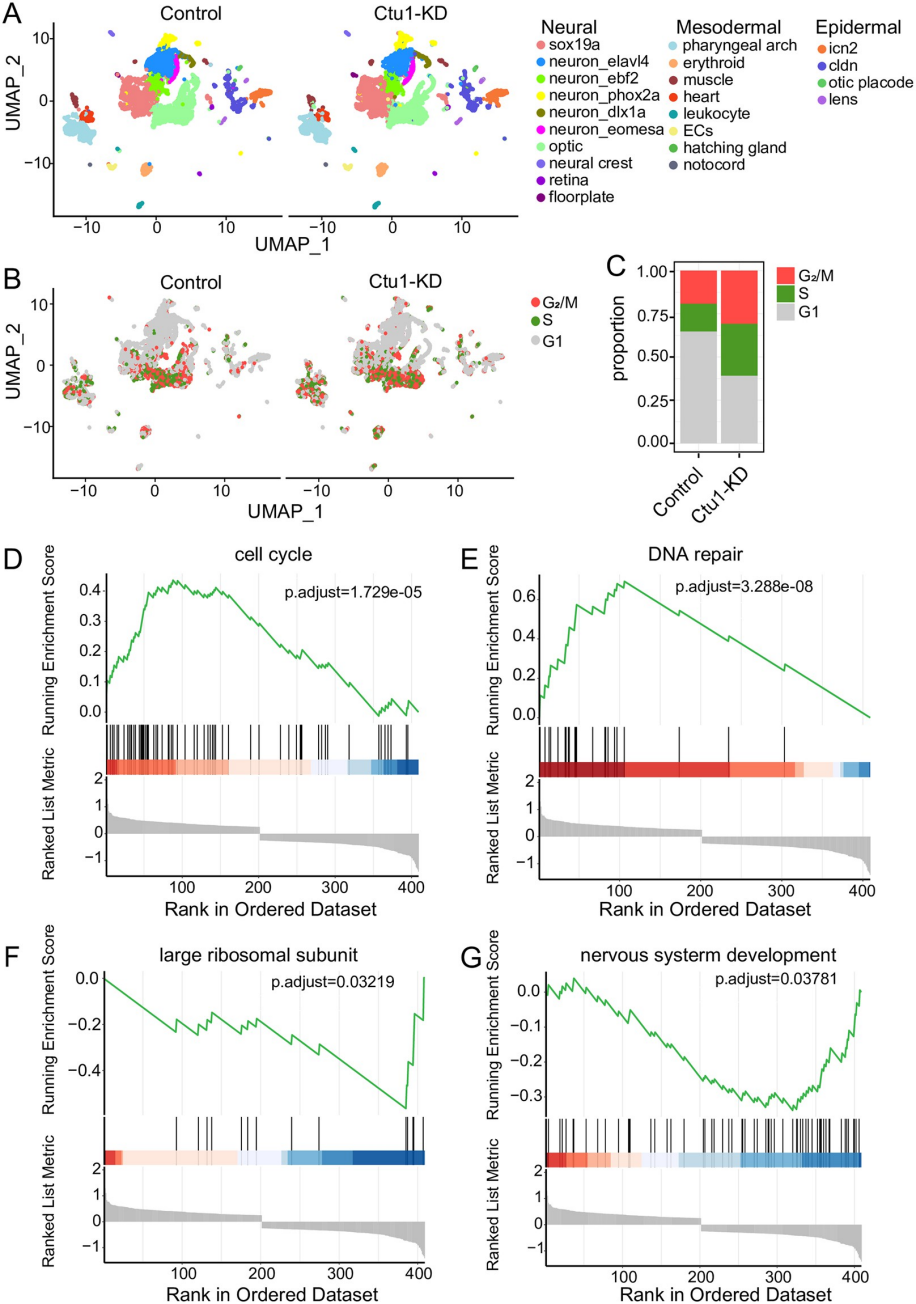
Single-cell transcriptome profiles of the *ctu1* morphant and control zebrafish embryos. UMAP visualization of all zebrafish cells, which are color-coded by cell type (A), and cell cycle phase (B). (C) The relative proportion of each cell cycle phase in the control and Ctu1 morphant. Gene sets of GSEA analysis shown are cell cycle (D), DNA repair (F), large ribosomal subunit (F) and nervous system development (G).

In order to create a transcriptome map of the zebrafish with *ctu1* KD, we used 20 zebrafish larvae at 2-dpf after MO microinjection from the control and *ctu1* morphant group for tissue digestion and scRNA-seq. In total, 20,788 cells were retained for subsequent analysis. Cells were clustered based on the gene expression profiles, and cell types were annotated based on the published literature and the CellMarker database [27, 28] (S2A and S2B Fig). UMAP visualization of a cluster analysis identifies 22 different cell types, which were both observed in the control and *ctu1* morphant group (Fig 3A). The *ctu1* morphant demonstrated a higher proportion of cells in the G_2_/M phase and S phase (Fig 3B and BC). We found 57 downregulated genes and 41 upregulated genes in the *ctu1* morphant samples. The upregulated genes are mainly enriched in cell cycle (Fig 3D) and DNA replication (Fig 3E), while the downregulated genes are predominantly enriched in the large ribosomal subunit (Fig 3F) and neuron system development (Fig 3G). These results suggest that *ctu1* deficiency induces DNA damage, thereby causing cell-cycle arrest at G_2_/M phase and activating DNA repair response.

### Heterogeneity analysis of the three main embryonic germ layers

**Fig 4:**
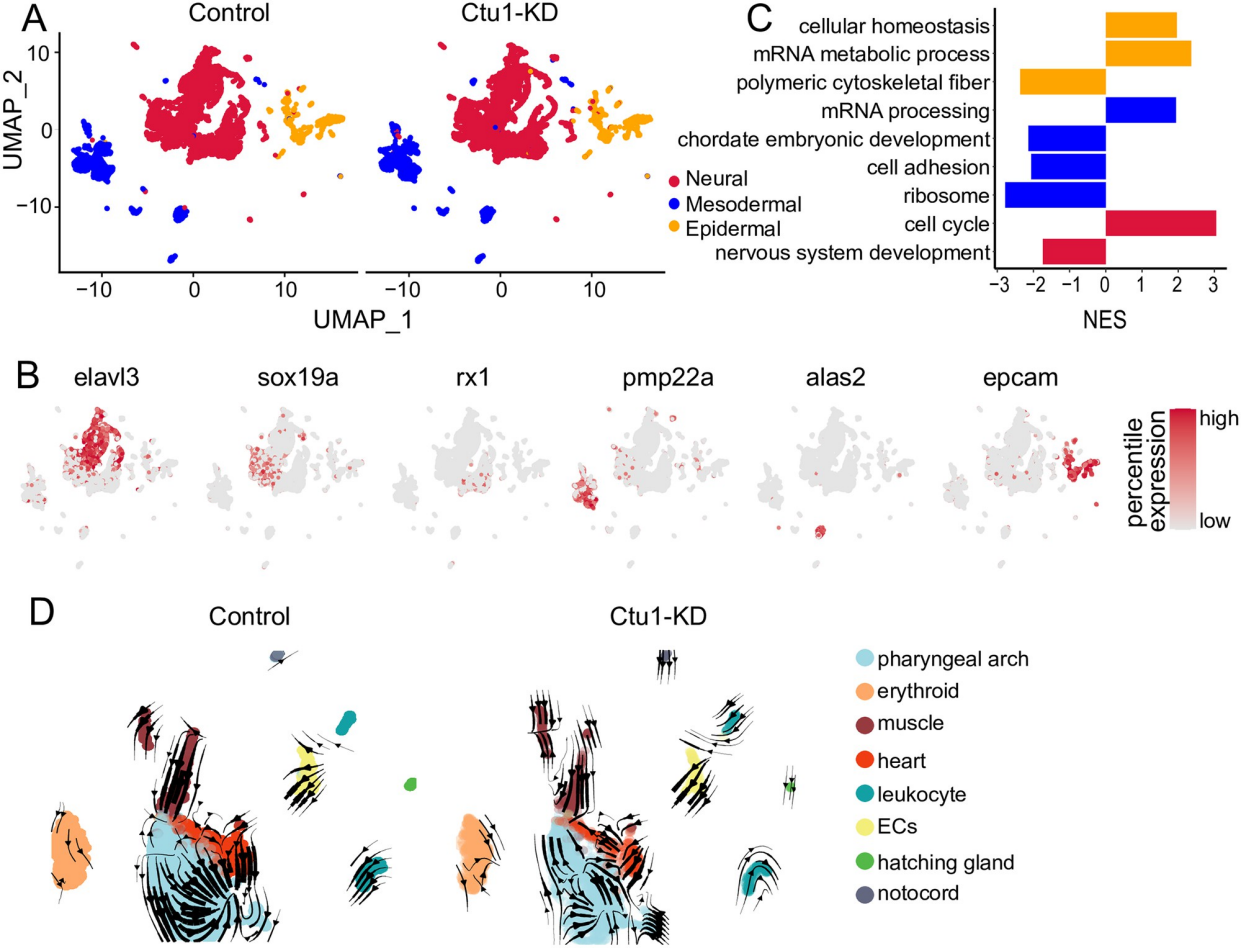
Comparative analysis of the control and *ctu1* morphant across three main embryonic germ layers. (A) UMAP visualization of three germ layers. (B) UMAP visualization of key marker genes expression. Color scale represents log-normalized expression. The marker genes for neural cells are *elavl3*, *sox19a*, *rx1*; for mesodermal cells, the markers are *pmp22a* and *alas2*; and for e pidermal cells, the marker gene is *epcam*. (C) The bar plot shows signal pathways affected by *ctu1* deficiency in epidermal, mesodermal, and neural cells as identified by GSEA. All terms demonstrate significant enriched (*adj*.*p* < 0.05) and normalized enrichment scores (NES) are shown. (D) RNA velocity plot of control and *ctu1* morphant mesodermal cells.

Three germ layers were isolated and DEGs analysis was conducted separately in the three germ layers-cells [27] (Fig 4A and 4B). For the epidermal cells, we identified four types, including the epidermal otic placode and three other cell subgroups marked by *icn2*, *cldn*, and *lens*, respectively (S3A Fig). GSEA analysis of the epidermal cells revealed that the absence of *ctu1* significantly upregulation of genes associated with cellular homeostasis and mRNA metabolic process pathways, while downregulation of genes related to polymeric cytoskeletal fibers (Fig 4C, yellow). The mesoderm cells were also sub-grouped clusters including pharyngeal arches, erythrocytes, muscles, heart, leukocytes, endothelial cells, hatching glands, and notochord (S3B Fig). GSEA results of the mesoderm cells indicated that genes involved in the mRNA processing pathway were significantly upregulated. In contrast, genes associated with chordate embryonic development, cell adhesion, and ribosome pathways were significantly downregulated in *ctu1* morphant (Fig 4C, blue). The neural cells were classified into the subgroups including the optic nerve, neural crest, retinal nerve, floorplate, and subpopulations marked by *sox19a*, *elavl4*, *ebf2*, *phox2a*, *dlx1a* and *eomesa* (S3C Fig). The GSEA analysis of neural cells demonstrated that a deficiency in *ctu1* resulted in a significant increase in genes associated with the cell cycle pathway and a significant decrease in genes related to the neural development pathway (Fig 4C, red). In summary, DEGs analysis in the three germ layers cells implied that *ctu1* plays a vital role in the mesodermal cells, while its impact on epidermal cells is not significant. We then conducted RNA velocity analysis on the mesodermal cells and observed that muscle differentiation primarily advances towards the pharyngeal arch and heart. Notably, the differentiation ability of the heart and pharyngeal arch was reduced in *ctu1* morphant (Fig 4D). Consequently, *ctu1* is essential for the normal growth and functional maintenance of the pharyngeal arch, heart and muscles.

### *ctu1* deficiency suppresses erythrocyte differentiation

**Fig 5:**
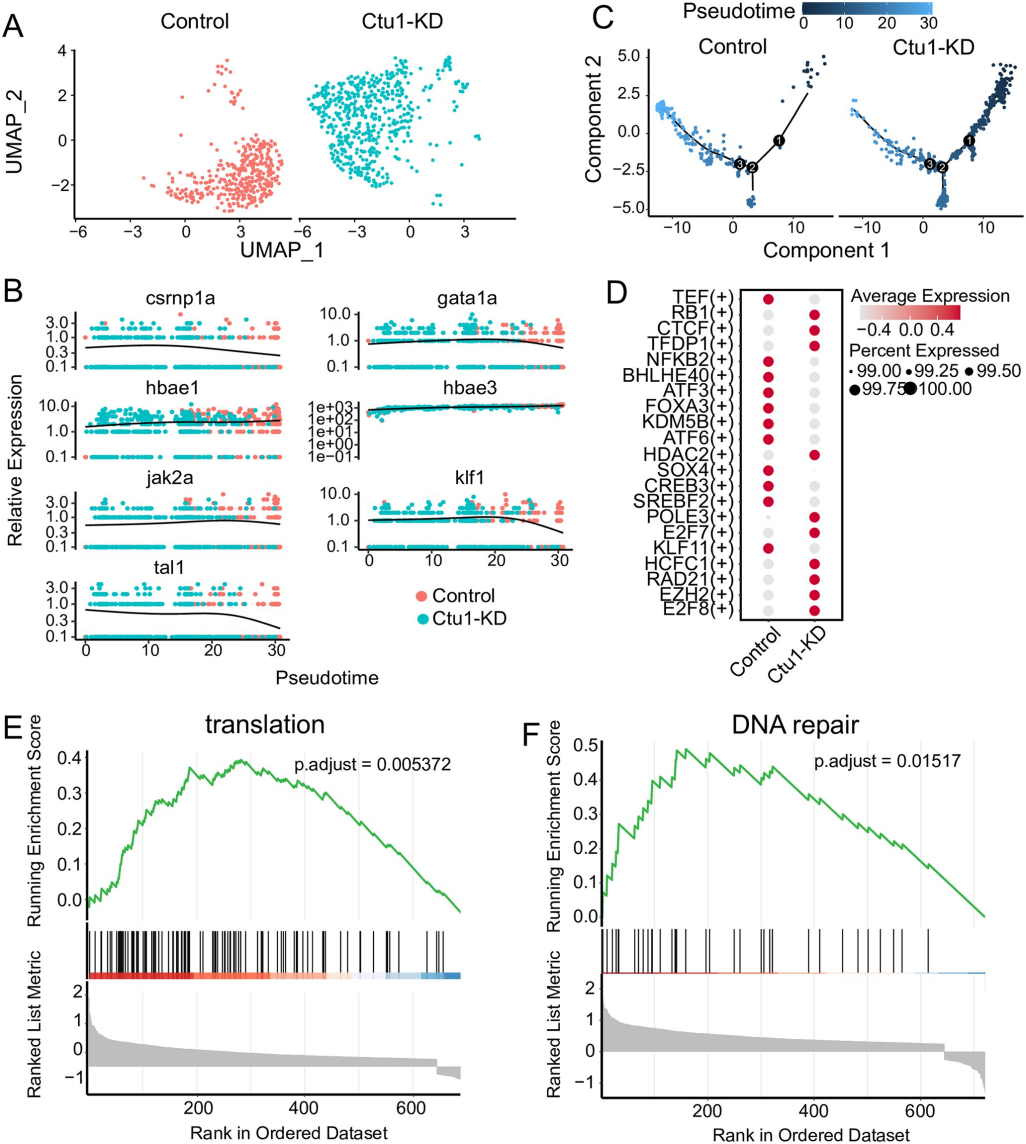
*ctu1* deficiency suppresses erythrocyte differentiation. (A) UMAP visualization of erythroid cells, colored according to samples. (B) The relative expression of the marker genes associated with erythroid differentiation in the pseudotime trajectories of control and *ctu1* morphant. (C) Pseudotime trajectories of erythroid cells. (D) Dot plots show changes in the expression of transcription factors across different samples. The color and size of circles indicate the average expression level and percentage of cells. GSEA analysis of control and *ctu1* morphant. Gene sets shown are translation (E), and DNA repair (F).

We extracted the erythrocyte populations of interest in our study for in-depth study. The transcriptome profiles of erythroid cells in the two groups were significantly different (Fig 5A). Previous research has established that *crnp1a*, *gata1a*, *jak2a*, *klf1* and *tal1* are necessary for the development of primitive hematopoiesis progenitors in zebrafish [29–33]. Primitive erythrocytes express embryonic globin genes (*hbae3* and *hbbe1*.*1*) [34]. We calculated the relative expression levels of these hematopoietic marker genes as they changed over time in two samples (Fig 5B). The results showed that *crnp1a*, *gata1a*, *jak2a*, *klf1*, and *tal1* were highly expressed in the *ctu1* morphant, with their expression decreasing in the control group as pseudotime changes. On the other hand, the expressions of *hbae1* and *hbae3* are ubiquitous, with the highest expression levels in control group. The results of the pseudotime analysis showed that *ctu1* morphant cells were distributed in the early stages of the differentiation trajectory (Fig 5C). Compared to the control, we identified regulators with high activity (Fig 5D), including *e2f7*, *e2f8*, *hcfc1*, and *hcfc2*, which play important roles in hematopoiesis [35, 36].*rb1*, *tedp1*, and *ezh2* affect the development of erythroid cells [37–40]. In contrast, the activity-regulating factor *klf11* [41], which is expressed in mature red blood cells, and the factors *srebf2*, *creb3*, and *atf6* involved in endoplasmic reticulum stress [42–44], as well as the *nfkb2* factor that plays a role in early differentiation of the bone marrow lineage [45], all show decreased activity. These results suggest that those specific regulons which are active in erythroid cells, exhibit difference between the control and *ctu1* morphants. GSEA results indicated that significant upregulated genes associated with translation (Fig 5E), and DNA repair (Fig 5F) pathways following *ctu1* gene silencing. These findings reinforce the heterogeneity of the two samples of erythroid cells and highlight that *ctu1* knockdown significantly inhibits erythroid differentiation.

### *ctu1* deficiency reduces the activity of the *angpt* and *notch* signaling pathways originated from the endothelial cells in the mesoderm

**Fig 6:**
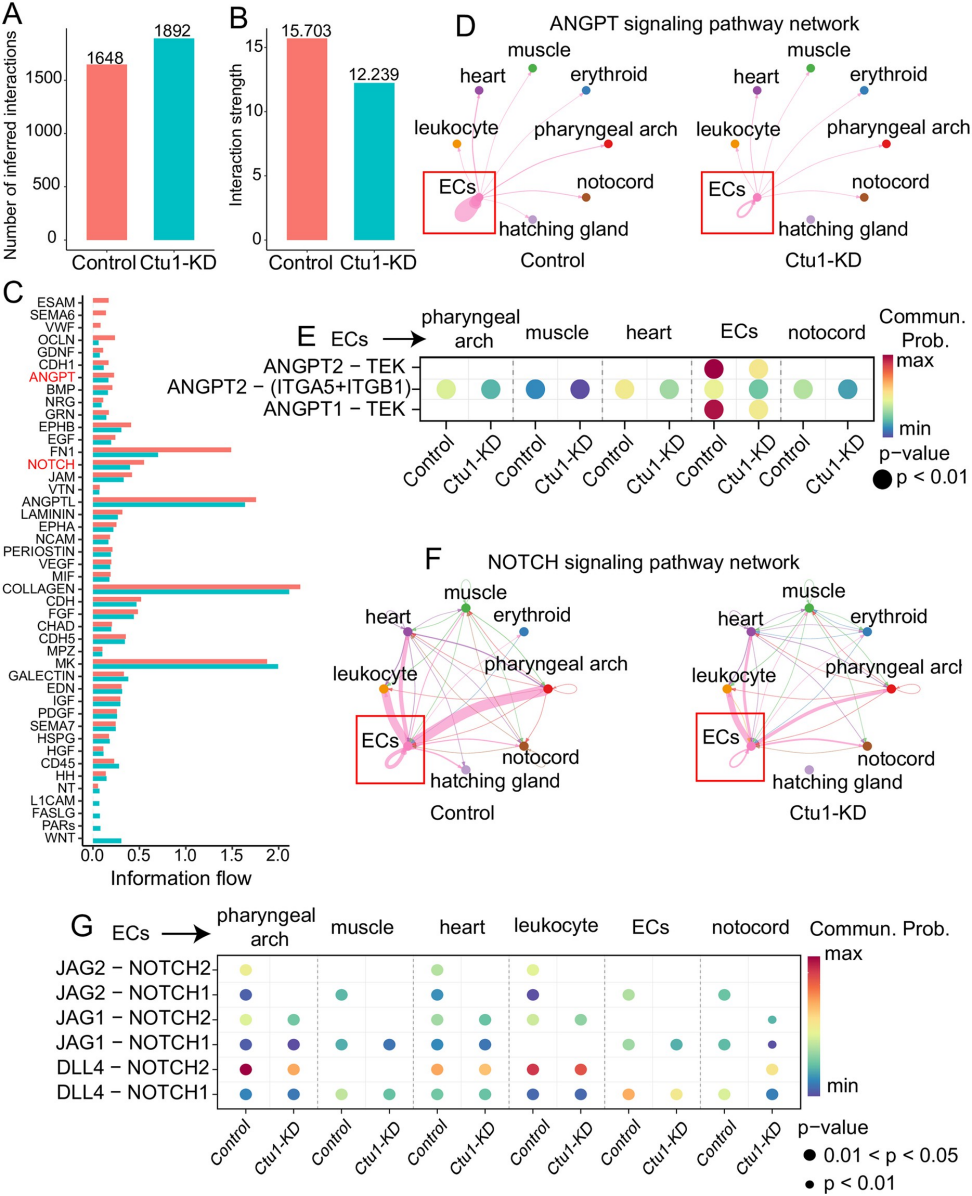
*ctu1* deficiency reduces the activity of the *angpt* and *notch* signaling pathways originated from the endothelial cells in mesoderm. Bar plot shows overview number (A) and strength (B) in control and *ctu1* morphant. (C) Ranking of active signaling pathways in control and *ctu1* morphant based on their overall information flow within the inferred cellular networks. Signaling pathways are colored according to condition where they are enriched. (D) The chord plot shows *angpt* signaling in sending and receiving cells. Nodes are colored by celltypes. The thickness of the line represents the strength of the signal. (E) Dot plots show communication probability of *angpt* signaling between endothelial cells (senders) and each celltypes (receivers). Blue, low communication probability; red, high communication probability. Size of circle represents the pvalue of cells with communication probability. Chord plots (F) and Dot plots (G) shows *notch* signaling pathway network.

To explore further insights into the communication network among mesoderm cells in *ctu1* morphant, we utilized the CellChat to infer the ligand-receptor pairs involved in interactions between different cell types. Initially, a comprehensive assessment of both the volume and intensity of cellular communication was conducted. The total communication volume with control and *ctu1* morphant increases, yet the overall communication intensity diminishes (Fig 6A and 6B). In particular, the intensity of several pro-angiogenesis signaling cascades, like *endothelial cell-selective adhesion molecule* (*esam*), *semaphorin 6A* (*sema6*), *Von Willebrand factor* (*vwf*), *occludin* (*ocln*), *and bone morphogenetic protein* (*bmp*), were significantly attenuated when Ctu1 was comprised (Fig 6C). In particularly, cell-cell communication showed that the strength of angiopoietin (*angpt*) signaling pathway network which originated from endothelial cells was significantly attenuated in *ctu1* morphant compared to the control (Fig 6D). The probability of *angpt-tek* communication was significantly attenuated in the *ctu1* morphant (Fig 6E). The *dll4*-mediated *notch* signaling pathway plays an important role in the occurrence and formation of embryonic blood vessels [46, 47]. Reduced levels of *dll4-notch* expression in zebrafish lead to abnormal endothelial cell numbers and exhibit ISV vascular defects [48, 49]. Here our data indicated that *dll4-notch* signaling which was predominantly derived from endothelial cells was significantly attenuated in pharyngeal arch, muscle, and endothelial cells (Fig 6F and 6G). In conclusion, the decreased autocrine *angpt-tek* and *dll4-notch* signalings in endothelial cells are probably responsible for the defective angiogenesis after *ctu1* suppression in zebrafish.

### Effects of CTU1 upon endothelial cells

**Fig 7:**
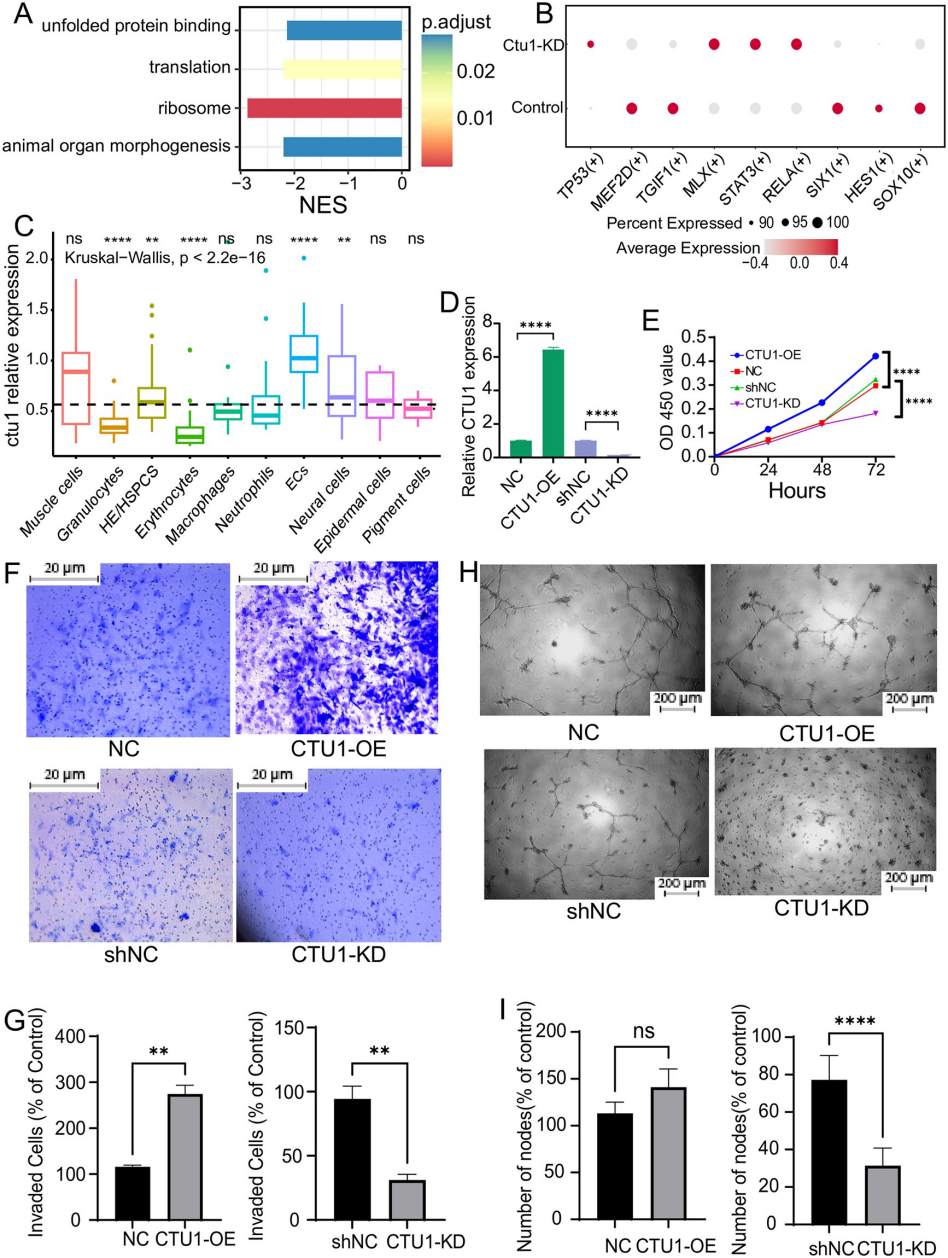
Single-cell data analysis of the endothelial cells in zebrafish, and the angiogenesis-related behaviors of human endothelial cells with differential expression of CTU1. (A) The bar plot shows signal pathways affected by *ctu1* deficiency in endothelial cells as identified by GSEA. (B) The dot plot illustrates the variation in transcription factor expression in control and *ctu1* morphant, with the color and size of the circles representing the average expression level and the proportion of cells, respectively. (C) The box plot illustrates the relative expression levels of *ctu1* of each cell type in 36-hpf zebrafish. ****, *p <* 0.0001. (D) The CTU1 expression levels of CTU1-KD and CTU1-OE HMEC-1 cells after exposing to the corresponding lentivirus. (E) The growth curve of CTU1-KD, CTU1-OE, and vehicle control HMEC-1 cells. Representative images (F) and quantifications of the migrated cells (G, n = 3 independent experiments) of HMEC-1 cells with different expression levels of CTU1 in the transwell assay. Scale bars represent 20 μm. Comparisons between each group were analyzed using Student’s t-test. **, *p <* 0.01. Representative images (H) and quantifications of the branch points (I, *n* = 3 independent experiments) of HMEC-1 cells with different expression levels of CTU1 in the tube formation assay. Scale bar, 200 μm. Comparisons between each group were analyzed using Student’s t-test. ****, *p* < 0.0001.

Endothelial cells play critical roles in angiogenesis. We performed DEGs analysis with the single-cell transcriptome data of the zebrafish endothelial cells (S3D Fig), and found that the decreased genes in the *ctu1* morphant was enriched in unfolded protein binding, translation, ribosome, and animal organ morphogenesis (Fig 7A). Furthermore, we conducted GRN analysis by utilizing pySCENIC to elucidate the transcriptional regulatory changes of *ctu1*. The results revealed that *ctu1*-deficient endothelial cells exhibited elevated activity levels of *tp53*, *mlx*, *stat3*, *rela* and decreased activity of *mef2d*, *tgif1*, *six1*, *hes1* and *sox10* (Fig 7B). The myocyte enhancement factor *mef2d* promotes tumor angiogenesis *in vitro* and *in vivo* and induces the expression of pro-antigenic cytokines in colorectal cancer cells [50]. *sox10* stem cells have the differentiate into perivascular cells, which play a crucial role in stabilizing newly formed microvessels [51].

By analyzing the GSE186423 dataset, which encompasses a total of 4,583 endothelial and hematopoietic progenitor cells from 36-hpf zebrafish embryos, we discovered that ctu1 exhibits the highest expression levels in zebrafish endothelial cells (Fig 7C). To confirm the effect of CTU1 on angiogenesis, we also applied human endothelial cells HMEC-1 *in vitro*, and transfected them with CTU1 over-expressing or silencing lentivirus to get CTU1-OE and CTU1-KD HMEC-1 cells (Fig 7D). We evaluated the cell growth, migration and tube formation ability of those HMEC-1 cells. Our results indicated that CTU1-OE significantly increased cell number, while CTU1-KD notably reduced cell viability after 72 hours’ culture (Fig 7E). In addition, the overexpression of CTU1 significantly enhanced the migratory capacity of HMEC-1 cells, and reducing CTU1 expression modestly decreased their migratory capacity (Fig 7F and 7G). In the tube formation assay, our experiments clearly showed that at the 16-hour time point, there were significant differences in tube branch length and total area covered among the CTU1-KD, CTU1-OE, and control HMEC-1 cells. In particular, CTU1-KD HMEC-1 cells failed to form vasculature branches (Fig 7H and 7I).

## Discussion

**Fig 8:**
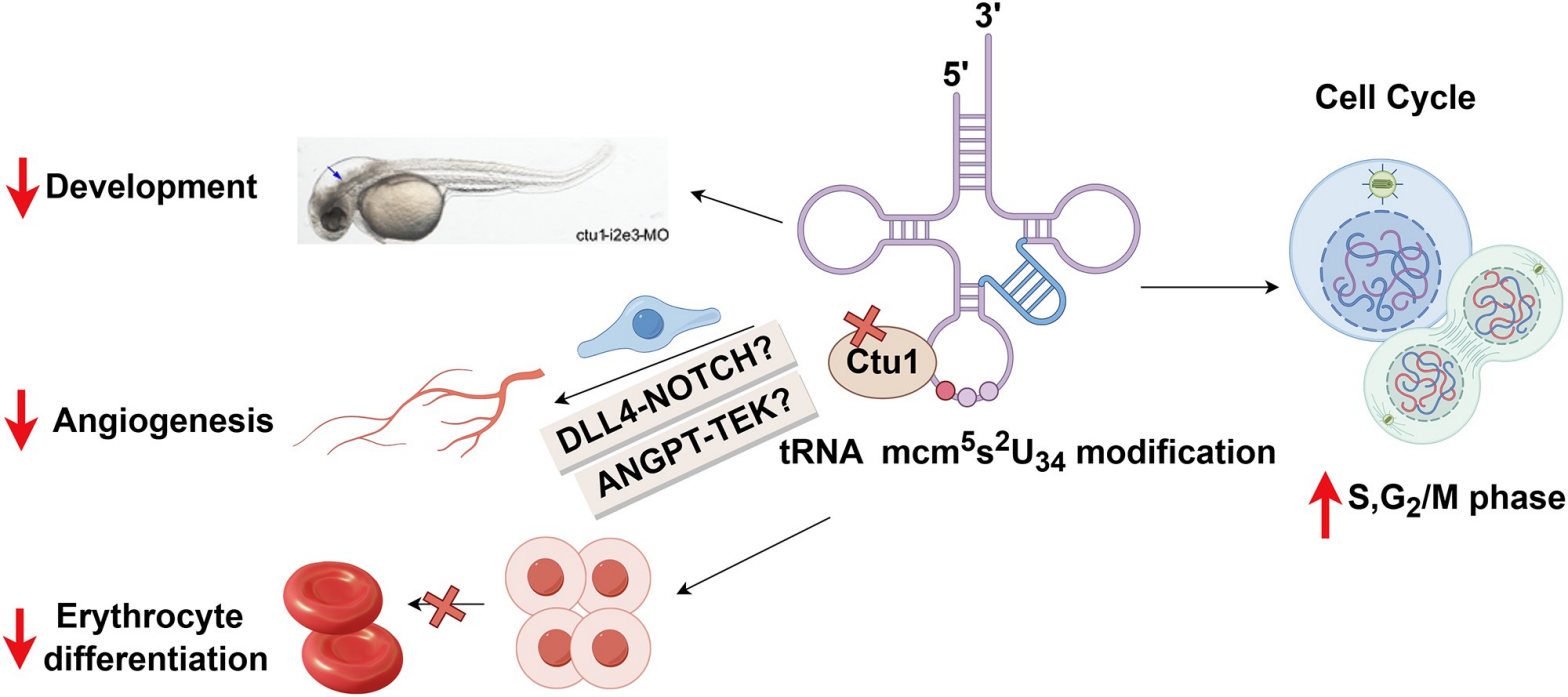
Model of the consequences of Ctu1 deficiency in zebrafish, figured by Figdraw. Recently, the essential role of mcm^5^s^2^U_34_ tRNA modification and their modifying enzymes in proliferation and development has been demonstrated from yeast to mammals [7]. For example, double deletion of the partner enzymes (Ctu1 and Elp3) is lethal to the cell in yeast [14]. Inactivation of the CTU complex leads to a thermosensitive decrease with aberrant development in the nematode and fission yeast [15]. In mice, a deficiency in Elp1 causes male infertility [52], while Elp3-KO embryos exhibits significant growth retardation and fails to develop beyond E12.5 [13]. Here our finding provides the first experimental evidence that s^2^U_34_ tRNA modifying enzyme CTU1 is essential in cellular proliferation and development in vertebrates.

In our study, we demonstrate that the conserved wobble uridine tRNA modifying enzyme CTU1 is critical for zebrafish embryo angiogenesis, development and differentiation. We show that *ctu1* deficiency leads to defects in cell cycle progression and nerve development, as well as the disturbed differentiation of nerve cell and erythrocyte. Endothelial cells are the most affected cell types when comprising Ctu1, with the decreased cell to cell communication of several pro-angiogentic signaling cascades, e.g. *esam5*, *bmp*, *ocld*, *dll4-notch* and *angpt-tek* (Fig 8). Our work is of great significance in revealing the vital role and the underlying mechanisms of CTU1 in angiogenesis, development and differentiation in vertebrates.

mcm^5^s^2^U_34_ tRNA modifications and their modifying enzymes are involved in human neurological diseases, and normal neurons development and generation of neurons as well [53]. Mutations in the six subunits of Elongator, and Alkbh8 is associated with human neurological diseases [9, 10, 54, 55]. Comprehensive transcriptome analysis in mouse embryos indicates that Elp1 is essential for the expression of genes responsible for nervous system development [56]. Elp1 and Elp2 mutation also causes a severe neurodevelopmental phenotype in mice models [12, 57]. Meanwhile, homozygous Ctu2 mutation was identified in patients diagnosed with a novel multiple congenital anomalies syndrome called DREAM-PL which is characterized by dysmorphic facies, renal agenesis, ambiguous genitalia, microcephaly, and lissencephaly [11, 58]. Here our analysis demonstrated that *ctu1* deficiency causes downregulation of genes in neuron system development, which provides further evidence and also the underlying molecular mechanisms that *ctu1* regulate neurological development in zebrafish.

Additionally, our analysis shows that the expression of the marker genes for early hematopoietic and mature erythrocyte in the control and *ctu1* deficient erythrocytes were separately distributed by pseudotime, and the *ctu1* deficient ones were mainly distributed in the early stages along the differentiation trajectory. The erythropoisis defects in *ctu1* deficient zebrafish embryo, together with the fact that loss of Elp3 causes bone marrow failure and compromises the grafting activity of hematopoietic stem cells [59], highlights the vital role of mcm^5^s^2^U_34_ tRNA modifications in hematopoisis.

We observed defective angiogenesis in *ctu1* morphant zebrafish larvae. During vertebrate embryogenesis, the pharyngeal arch artery connects the heart to the dorsal aorta, and defects in pharyngeal bursa development are commonly associated with abnormal vascular development [60]. RNA velocity analysis reveals reduced flow flux of muscle-pharyngeal arches in the *ctu1* deficient embryo, which maybe responsible for the observed vascular defects. Furthermore, cell communication analysis indicates that several pro-angiogenesis genes, like *esam* [61], *sema6a* [62], *ocln*, *bmp*, *fn1*, *angpt* [63], and *notch* [46, 47] are all reduced in the mesodermal layer of the *ctu1* morphant zebrafish. Their critical roles in neovascularization and angiogenesis have been well documented. *angpt* and *tek* serve as major regulators of angiogenesis in both physiological and pathologic conditions [64]. *ocln* is a functional marker of vascular endothelial cells on tube-forming activity plays, and plays a critical role in tube formation, sprouting, and proliferation [65]. *fn1* and *bmp* signaling has been shown to be important for angiogenesis [66]. Reduced levels of *dll4-notch* expression in zebrafish lead to abnormal endothelial cell numbers and exhibit ISV vascular defects [48, 49], which was similar with our observation in the transparent zebrafish emybro after *ctu1* deficiency. In summary, the reduced communication of those pro-angiogenic genes and signaling cascades, and the defective angiogenesis phenotype, identifies *ctu1* as a novel regulator for angiogenesis and neuovasculogenesis in zebrafish.

Furthermore, as *ctu1* is highly expressed in endothelial cells, and endothelial cells are hot target after *ctu1* deficiency by bioinformatics analysis. We further analyzed the transcriptome data of those endothelial cells, and found that *ctu1* knockdown significantly inhibited the expression of ribosomal genes in endothelial cells. Ribosomes are the main effector of the translational machinery to synthesize proteins. Decrease of the ribosomal subunit-selective homeostasis and inhibition of the translation process has been documented to be decreased with reduced tRNA biosynthetic activity [67]. The existence of a connection between tRNA modification biology and proteins in the ribosome has been shown in S. pombe and S. cerevisiae [68]. Here our analysis data also links mcm^5^s^2^U_34_ tRNA modifying enzyme Ctu1 to ribosomal gene expression, and the biological function of endothelial cells.

In conclusion, this is the first study which implies that cytosolic thiouridylase CTU1 is essential for angiogenesis and embryonic development. Our discovery indicates that the epigenetic modification of tRNA-U_34_, especially s^2^U_34_ modification and its modifying enzymes, are key regulators of angiogenesis and differentiation in vertebrates.

## Supporting information

S1 Fig Bright field and EGFP fluorescence images of each group of representative zebrafish. (A and B) Bright-fieldand EGFP fluorescentimages depict the overall morphology of control and *ctu1* morphant at 2-dpf. (C) Urm1-targeted MO design strategy. (D) PCR analysis of control and *urm1* morphant. (E and F) Bright-fieldand EGFP fluorescentimages depict the overall morphology of control and *urm1* morphant at 2-dpf. Quantification of the number of complete ISVs (G) and CVP (H). Columns, mean; bars, SEM (n = 10; unpaired student’s t-test; ***, *p <* 0.001).
(TIF)

S2 Fig Single-cell transcriptome profiles of the *ctu1* morphant and control zebrafish embryos. (A) UMAP visualization of zebrafish cells, which are colored by clusters. (B) The violin plot shows the expression of top marker genes in each cluster.
(TIF)

S3 Fig Single-cell transcriptome profiles of three germ layers from ctu1 morphants and control zebrafish embryos. (A) UMAP visualization of epidermal cells, which are colored by cell types. (B) UMAP visualization of mesoderm cells, which are colored by cell types. (C) UMAP visualization of neural cells, which are colored by cell types. (D) UMAP visualization of endothelial cells, which are colored by clusters.
(TIF)

S1 Raw image (TIF)
