# Supplementary figures and images for: The conserved wobble uridine tRNA thiolase Ctu1 is required for angiogenesis and embryonic development
Source: PLoS One. 2024 Dec 20;19(12):e0315854. doi: 10.1371/journal.pone.0315854 (PMC11661634; doi:10.1371/journal.pone.0315854)

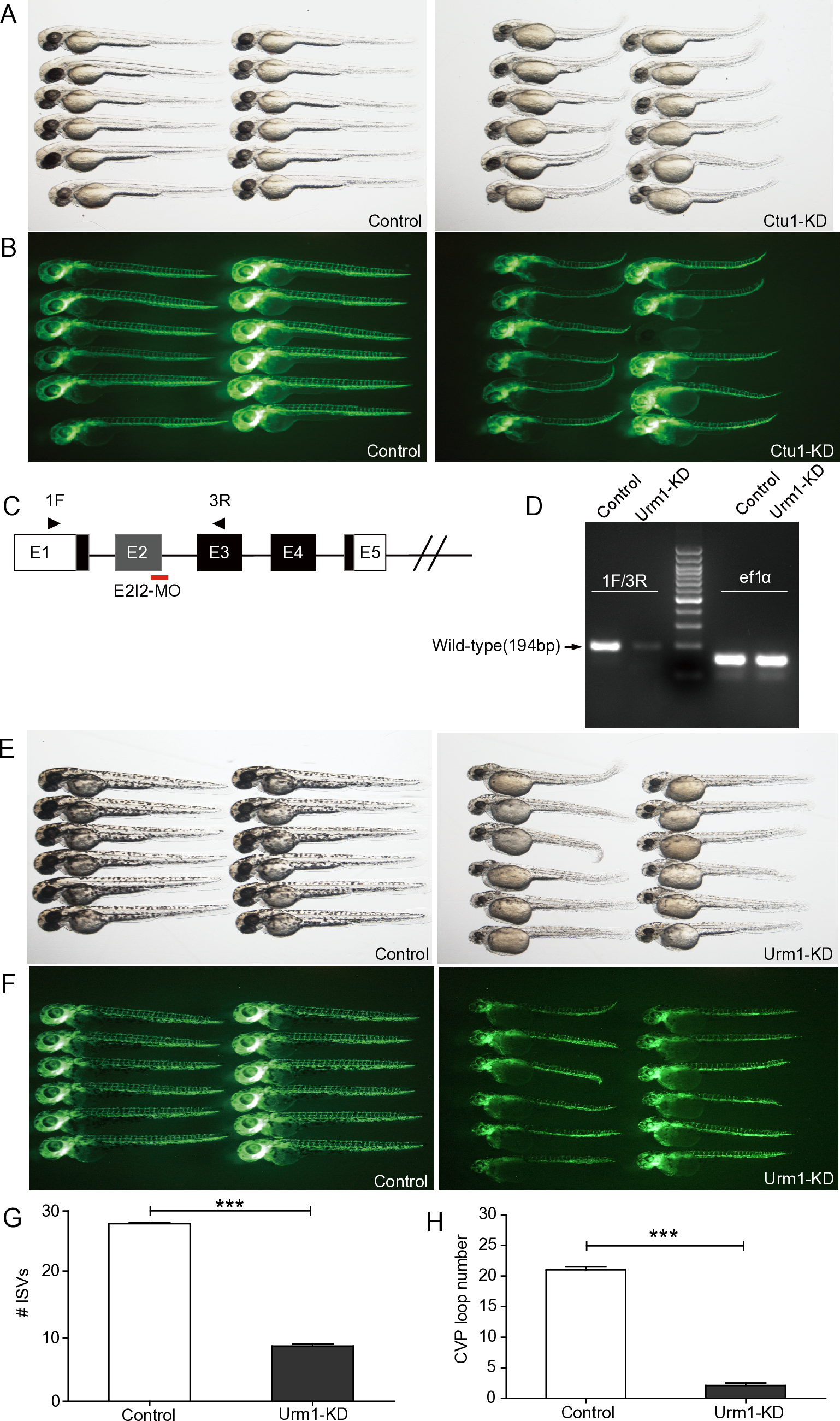

Supplement: S1 Fig — (A and B) Bright-fieldand EGFP fluorescentimages depict the overall morphology of control and ctu1 morphant at 2-dpf. (C) Urm1-targeted MO design strategy. (D) PCR analysis of control and urm1 morphant. (E and F) Bright-fieldand EGFP fluorescentimages depict the overall morphology of control and urm1 morphant at 2-dpf. Quantification of the number of complete ISVs (G) and CVP (H). Columns, mean; bars, SEM (n = 10; unpaired student’s t-test; ***, p < 0.001). (TIF) [file pone.0315854.s001.tif]

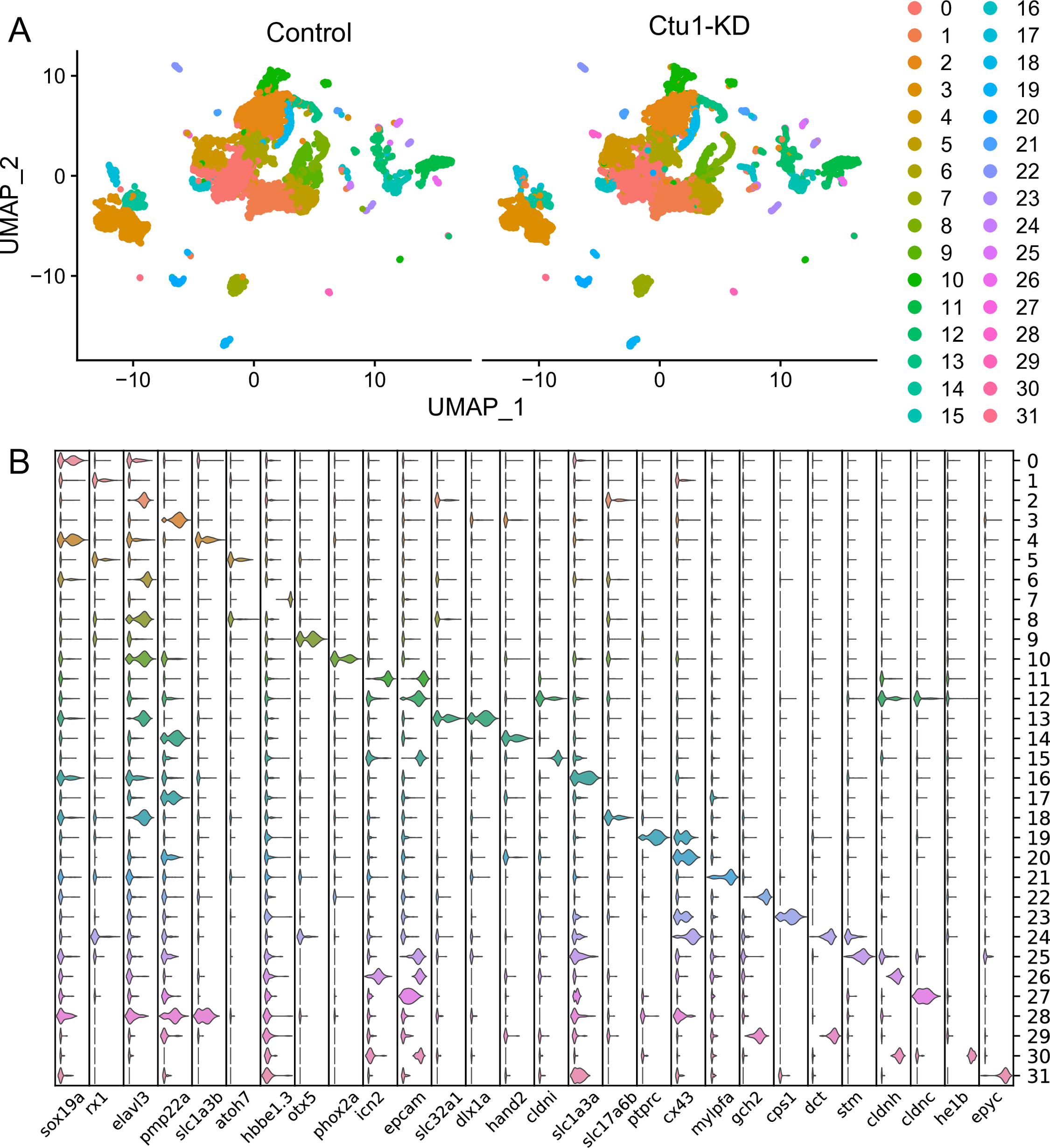

Supplement: S2 Fig — (A) UMAP visualization of zebrafish cells, which are colored by clusters. (B) The violin plot shows the expression of top marker genes in each cluster. (TIF) [file pone.0315854.s002.tif]

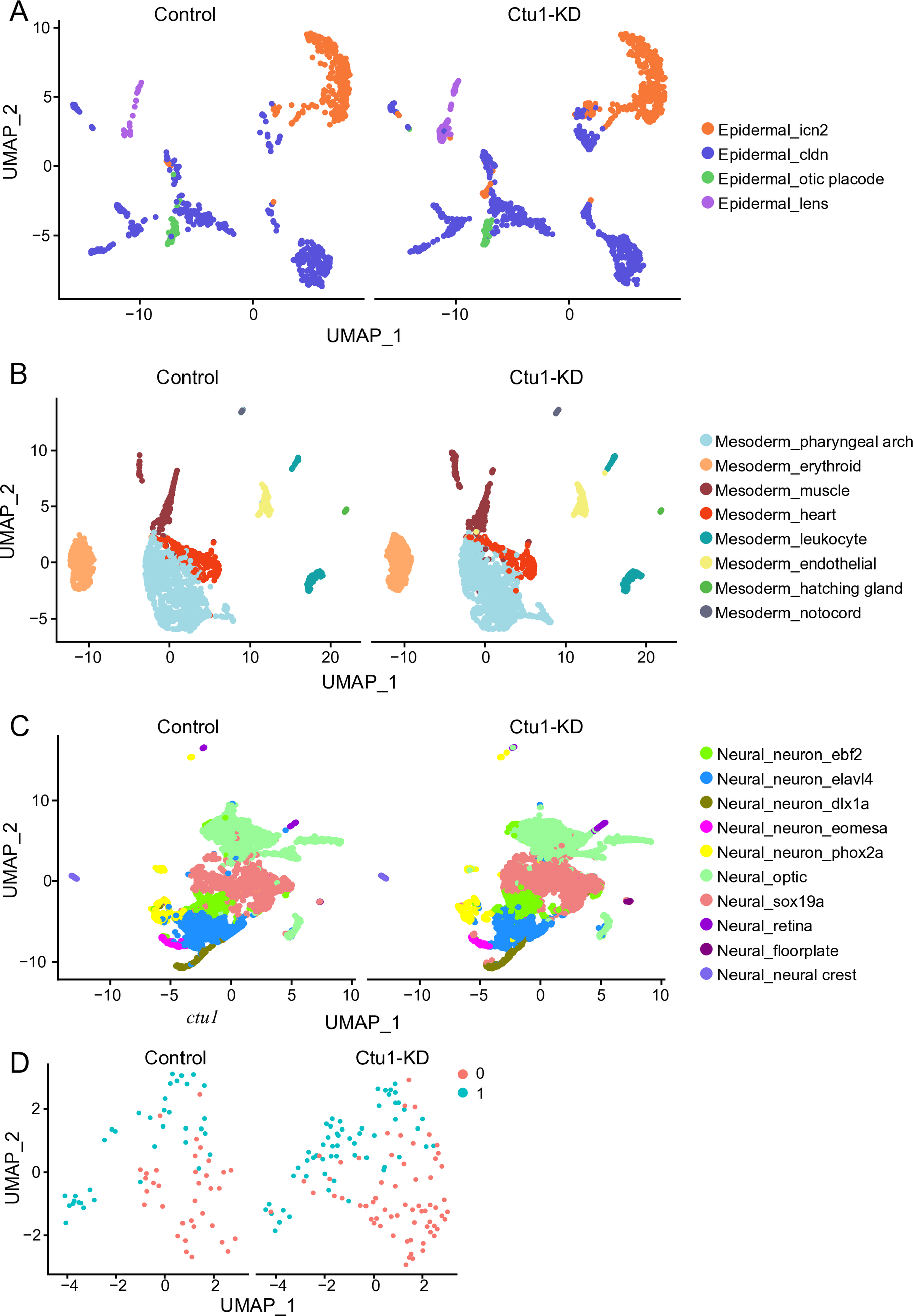

Supplement: S3 Fig — (A) UMAP visualization of epidermal cells, which are colored by cell types. (B) UMAP visualization of mesoderm cells, which are colored by cell types. (C) UMAP visualization of neural cells, which are colored by cell types. (D) UMAP visualization of endothelial cells, which are colored by clusters. (TIF) [file pone.0315854.s003.tif]

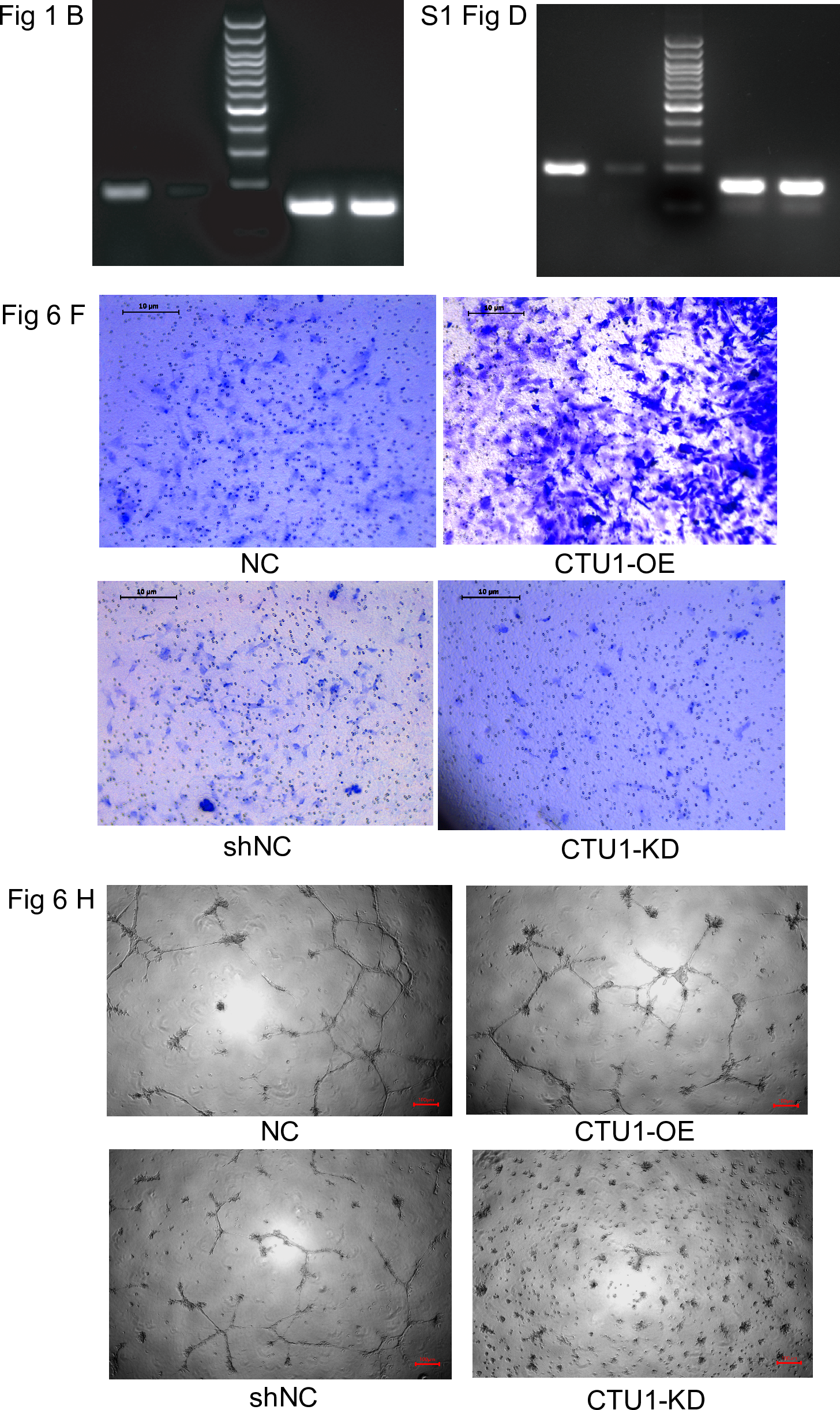

Supplement: S1 Raw image — (TIF) [file pone.0315854.s004.tif]
